# Antenatal care packages with reduced visits and perinatal mortality: a secondary analysis of the WHO antenatal care trial - Comentary: routine antenatal visits for healthy pregnant women do make a difference

**DOI:** 10.1186/1742-4755-10-20

**Published:** 2013-04-12

**Authors:** G Justus Hofmeyr, Ellen D Hodnett

**Affiliations:** 1 Effective Care Research Unit, University of the Witwatersrand/Fort Hare, Eastern Cape Department of Health, Eastern Cape, South Africa; 2 Lawrence S. Bloomberg Faculty of Nursing, University of Toronto, Toronto, Canada

## Abstract

The practice and timing of routine antenatal visits for healthy pregnant women, introduced arbitrarily and without evidence of effectiveness, have become entrenched in obstetric practice over the last century. In 2001 the large, cluster randomized WHO Antenatal Care Trial concluded that a goal-orientated package of antenatal care with reduced visits seemed not to affect maternal and perinatal outcomes. The reduced visit package has been implemented in several countries. The current re-analysis finds that the significantly increased perinatal mortality which occurred in the reduced visit package persists after adjustment for potential confounding factors. The WHO Antenatal Care Trial provided the first evidence from a randomized trial that the traditional high frequency of routine visits in the third trimester may well reduce perinatal mortality.

## 

Routine antenatal care visits for healthy pregnant women were introduced in Europe [1] and North America [2] almost a century ago on the unproven assumption that they would improve outcomes for mother and baby. Repeated visits by healthy women to a health facility represent a substantial intrusion in women’s daily lives, and it would be useful to have evidence of benefit with which to justify our expectations of compliance. The practice has become so entrenched that randomized trials of antenatal care versus no antenatal care are unlikely to be undertaken.

In 2001 the results of a landmark trial comparing a package of antenatal care with reduced, goal-orientated visits versus standard antenatal care were published in The Lancet [3]. Although perinatal mortality was increased in the reduced visit package (234/11672, 2.0% versus 190/11121, 1.7%), the conclusion arrived at by the authors was that provision of antenatal care by the new model seemed not to affect maternal and perinatal outcomes.

This paper and derivative publications such as the WHO manual for the implementation of the new model (http://www.who.int/reproductivehealth/publications/maternal_perinatal_health/RHR_01_30/en/index.html) have impacted on antenatal care practice in low-income countries such as Thailand [4], South Africa (http://www.doh.gov.za/docs/stratdocs/2012/MNCWHstratplan.pdf) and possibly Nigeria [5].

The authors of the current paper are to be commended for undertaking this re-analysis of the WHO antenatal care trial data. The crude risk ratio (95% confidence interval) for the perinatal mortality rates indicated above was 1.20 (1.04, 1.38). After adjustment for potential confounding factors, the risk ratio remained significantly increased at 1.18 (1.01, 1.37). They point out that the increased risk of fetal death between 32 and 36 weeks gestation could be due to reduced number of visits, heterogeneity in study populations or differences in quality of care. The fact that perinatal mortality was not a primary outcome of the trial increases the possibility that the ‘statistically significant’ difference in perinatal mortality might have occurred by chance. However, given that this was a large, well-designed cluster randomized trial (24 000 women studied in 53 randomized clusters) which sought to minimize the possibility of confounding factors, that the current re-analysis was robust after adjustment for potential confounding factors, and that the increase in perinatal mortality is consistent with trends in the two other cluster randomized trials conducted in Zimbabwe [6], we find the evidence, that a reduced number of antenatal visits is associated with increased perinatal mortality, compelling.

A link between number of antenatal visits and perinatal mortality is certainly plausible. Asymptomatic conditions such as pre-eclampsia, fetal growth restriction and unexplained intrauterine death may present unexpectedly in the third trimester, and an increased number of routine visits may detect these conditions earlier, or elicit a report of reduced fetal movements earlier, allowing more timely intervention.

This paper provides sound information to guide the decisions of policymakers regarding the number of antenatal visits which should be offered with the available resources. The importance of the content of routine antenatal care should not be lost, when decisions are made about numbers of visits. The two are (or should be) inextricably linked, as they were in the original trial report [3].

Most importantly, after a century of blind faith, this paper provides probably the first direct evidence from a randomized trial that routine antenatal visits for healthy pregnant women do make a difference.
